# The effect of telenursing training based on family-centered empowerment pattern on compliance with diet regimen in patients with diabetes mellitus type 2: a randomized clinical trial

**DOI:** 10.1186/s12902-022-00953-4

**Published:** 2022-02-09

**Authors:** Negar Shahabi, Mitra Kolivand, Nader Salari, Parvin Abbasi

**Affiliations:** 1 Department of Nursing, Student Research Committee, Kermanshah University of Medical Sciences, Kermanshah, Iran; 2 Department of Midwifery, School of Nursing and Midwifery, Kermanshah University of Medical Sciences, Kermanshah, Iran; 3 Department of Biostatistics, School of Nursing & Midwifery, Kermanshah University of Medical Sciences, Kermanshah, Iran; 4 Department of Nursing, School of Nursing and Midwifery, Kermanshah University of Medical Sciences, Kermanshah, Iran

**Keywords:** Telenursing, Family-centered empowerment pattern, Compliance, Diabetes type 2, Randomized clinical trial

## Abstract

**Background:**

Telenursing facilitates access to efficient care and acceptance and compliance with treatment at home. Given wide complications of lack of compliance with treatment in causing complications and progression of diabetes and role of the family in attending the patient, this study aimed to investigate the effect of telenursing training based on family-centered empowerment pattern on compliance with diet regimen in patients with diabetes mellitus type 2.

**Methods:**

This was a randomized controlled clinical trial. The study population was patients with diabetes mellitus type 2 referred to Alzhara hospital at Gilan Gharb in 2019, of which 60 individuals out of them were classified randomly into two groups of intervention and control. Eight 30-min sessions of family-centered training were held through telenursing for the intervention group. Data were gathered before and after the intervention by standard questionnaire of Mudanlo in both groups and was analyzed using SPSS software version 22.

**Results:**

There was no significant difference among the two intervention and control groups before the study regarding demographic variables (*p* > 0.05). The scores of subscales of making effort for treatment, intention to take the treatment, adaptability, integrating illness into life, stick to the treatment, indecisiveness for applying treatment, and total score of compliance were significantly increased after training intervention (*p* = 0.001).

**Conclusions:**

Results of the study indicates positive effects of performing family-centered empowerment pattern using telephone call follow-up on increasing compliance with diet regimen in patients. Therefore, it is recommended to perform family-centered patterns in health policy-makings and also hospitals and other diabetic patients.

## Background

Diabetes type 2 is a metabolic disorder that is identified for high blood sugar in insulin-resistance status and partial deficiency of insulin [1, 2]. The rate of incidence of diabetes is increased considerably in recent 50 years and made it the fifth leading cause of mortality in the world, the fourth common cause of referral to a physician, and the biggest epidemics of the century [3–5].

Despite wide complications of the disease such as cardiac diseases, stroke, diabetic retinopathy, organ amputation [6], its serious complications and disabilities could be prevented through appropriate control and care [7]. The diabetic patient or his/her associates should supervise blood glucose and this supervision requires training [8]. Training in the first stages of diagnosis and using self-care approaches have the main role in nursery care [9]. About 50% of diabetic patients might make mistakes in skills related to self-care. They might forget some information or cannot learn them, and also emotional factors might reduce the ability of the patient to perform daily care efforts [10].

In this regard, receiving support from family makes motivation and improvement of self-care behaviors in diabetic patients [11]. Accordingly, the concept of family-centered empowerment in chronic patients is considered [10]. The main objective of this pattern is to empower the family system (patient and other members of the family) to promote the level of health. The process of family-centered empowerment improves the quality of life, responsibility, better interaction with health care providers, satisfaction, better response to treatment, prevention of complications, reducing therapeutic costs, and positive attitude on disease [12, 13]. In family-centered empowerment, the active presence of the family plays an important role in assessing and identifying patients’ needs [11]. Many problems at home occur due to the lack of awareness of the patient and his family about patient care, which is due to lack of access to the center or a source to answer their questions [14].

Mobile communications make opportunities for care to be transformed to the living place of the patients daily. The research showed that patients are most willing for telephone consultation by their physicians, and telephone services are the most effective and cost-effective method to follow in chronic diseases [15–17]. Nowadays, through progress in technology, telenursing method (using information technology and long-distance communication in the nursery) through providing care using communication instruments such as the internet, telephone, video-call, etc. can facilitate access to effective care in-home, reduce costs, and improve relations among the client and nurse, reduce repetitive examinations, eliminate obstacles related to time and place, improve relations among patient, patient’s associates, and care providers and life quality of patients due to access to necessary and vital information in necessary situations [16]. In addition, telenursing reduces referrals to the emergency unit and also increases relations among patients, their associates, and nurses. Telenursing help client and her/his family to perform required cares at home actively with greater knowledge and awarenss and adhere the prescribed therapeutic program [17, 18]. Education and counseling increase the self-care of diabetic patients and can provide information to patients and their families through telenursing and provide an opportunity to answer their questions at home [19, 20]. The studies conducted in recent decades indicate that telenursing is effective in the improvement of outcomes of diseases such as asthma, myocardial infarction, cancer, diabetes and Alzheimer’s disease [15].

Due to the role of telenursing in family-centered training and importance of family in compliance with diet regimen by the patients, this study aimed to investigate the effect of training of telenursing based on family-centered empowerment on Compliance with diet regimen in patients with diabetes mellitus type 2.

## Methods

### Study design and setting

The current study was a randomized clinical trial through pre and post test assessment with clinical trial code (IRCT20130812014333N115) (first registration in 06/03/2019) and assessed the effect of telenursing training based on family-centered empowerment by Mudanlo questionnaire of compliance with diet regimen among patients in diabetes Clinic at ALzahra Hospital of Gilan Gharb in the west of Iran.

### Participants

**Fig. 1:**
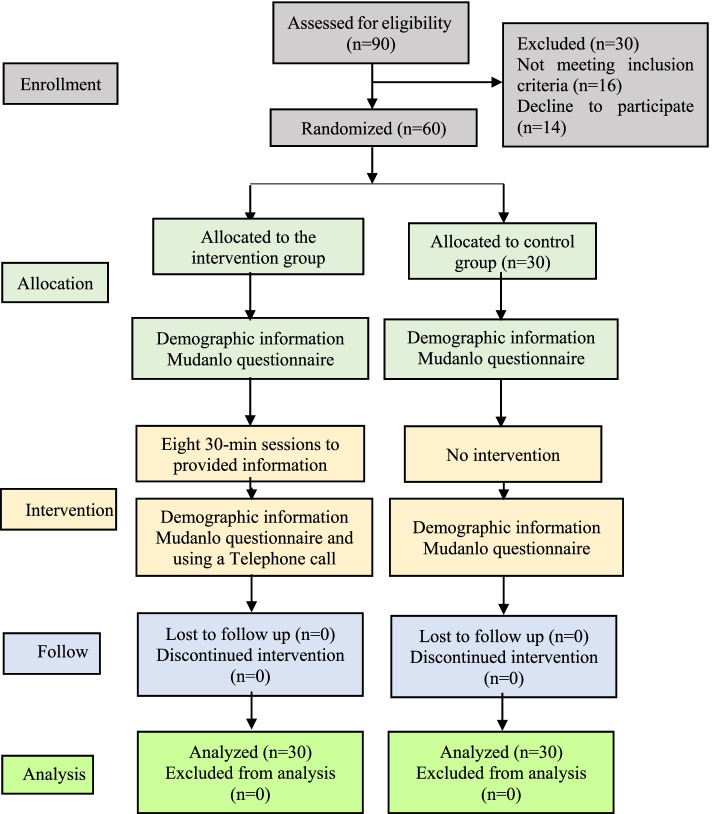
Consort flow diagram of the study

The study population consists of patients with diabetes mellitus type 2 referred to diabetes clinic at Alzahra Hospital of Gilan Gharb, Kermanshah, Iran. The sample size was computed by considering α = 95%, and β = 90% and based on the study by Razmaraei et al., [21] by considering 10% attrition rate at 30 individuals in each group (in total 60 individuals). Individuals were selected through a convenient method and their allocation into two groups was done through randomizing; so that 60 consecutive numbers were written on paper separately and the papers were located in the container. Selected even numbers were allocated to group A (control), and selected odd numbers were allocated to group B (intervention). To match several selections in groups, the picked numbers were not returned to the container. Inclusion criteria were as follow: definite diagnosis of diabetes mellitus type 2 by the physician, aged between 30 and 55 years old, passing at least 6 months from the diagnosis of diabetes, living with family, patient or one family member should have literacy, lack of serious and disabling complications caused by diabetes, lack of addiction, not using psychotropic drugs, not being participated in other similar research. Exclusion criteria include lack of satisfaction of the individual and the family to continue participating in the study, lack of responding and cooperating of the active family member. Before performing the study, the objectives were explained to each participant and they were assured that their information will be remained confidential and written informed consent was obtained from all of them (Fig. 1).

### Instruments

Data was gathered by standard questionnaire of compliance with diet regimen by Mudanlo and demographic information form. Mudanlo questionnaire consists of 40 items in seven aspects of making effort for the treatment (9 items), intention to take the treatment (7 items), adaptability (7 items), integration illness into life (5 items), stick to the treatment (4 items), commitment to the treatment (5 items), and indecisiveness to the treatment (3 items), and its scale if 5 grade Likert. This questionnaire has face, content and construct validity and is localized. Internal consistency of the questionnaire was achieved by computing Cronbach’s alpha of 0.92 and reliability of stability of the questionnaire was achieved by performing a retest with an interval of 2 weeks (*r* = 0.87) [22].

### Procedures

In this research, after obtaining permission from the ethics committee (KUMS.REC.1397.1010), the researcher referred to the clinic of diabetes of Alzahra hospital of Gilan Gharb to provide samples from patients though obtaining a cover letter from the deputy of research and postgraduates of the Kermanshah University of Medical Sciences. Firstly, the researcher held a face-to-face session in the hospital to gather information and justify the progress of research and compliance with diet regimen questionnaire was filled before the study by the patients. Then the researcher contacted individuals in the intervention group (patient and any member of the family) for eight 30-min sessions and provided information for the patient and the family member on diet therapy. Telephone call was held for the intervention group twice a week until the end of the study. At the end of the 8 weeks, Mudanlo questionnaire was provided for individuals in the intervention and control group and the changes in compliance with diet regimen were assessed in study groups.

### Data analysis

SPSS software version 22 was used to analyze data. Quantitative variables were expressed as mean and qualitative variables were expressed as frequency (number and percentage). The normality of distribution of the quantitative data was assessed using Kolmogrov-Smirnov test. To compare demographic characteristics and compare the two groups before and after the intervention based on being or not being, normal Yates Correction Test, Chi-Squared test, Mann-Whitney U, Wilcoxon Test, Paired-samples t-test, Independent t-test was used.

## Results

The patients of the control and intervention groups were homogeny regarding personal characteristics. Out of 60 patients, the majority of patients were married, women, employees, had a diploma and associate degree with an income below 1.5 million tomans monthly and mean age of 45 years old (Table 1).

**Table 1:** Comparison of demographic information of two intervention and control groups

Variable	Dimensions	Intervention group. n (%)	Control group n (%)	*P*.value
Gender	Male	14 (51.9)	13 (48.1)	0.50^a^
	Female	16 (48.5)	17 (51.5)	
Education	Under diploma	8 (50)	8 (50)	0.50^b^
	Diploma and Associate Degree	13 (48.1)	4 (51.9)	
	Bachelor’s degree and MA	9 (52.9)	8 (50)	
Job	Housewife	8 (50)	8 (50)	0.95^b^
	Employee	13 (48.1)	14 (51.9)	
	Other	9 (52.9)	8 (47.1)	
Income (million Tomans)	< 1.5	17 (51.5)	16 (48.5)	0.80^b^
	1.5-2.5	5 (41.7)	7 (58.3)	
	> 2.5	8 (53.3)	7 (46.7)	
Marital Status	Single	8 (47.1)	8 (52.9)	0.77^a^
	Married	22 (51.2)	21 (48.8)	
Age	Mean ± SD	45.9 ± 7.1	42.6 ± 6.8	0.07^c^
BMI	Mean ± SD	28.8 ± 3.4	28.2 ± 3.5	0.18^d^
The years of having diabetes	Mean ± SD	4.1 ± 1.6	3.6 ± 1.4	0.39^c^

*P*.value significant at *P* < 0.05

*SD* Standard deviation

^a^Yates Correction Test

^b^Chi-Squared test

^c^Independent t-test

^d^Mann-Whitney U

Results showed that there was no significant difference among two control and intervention groups before intervention regarding score of compliance with diet regimen and its all aspects (*p* > 0.05). But, after intervention of family-centered empowerment, a significant difference was observed in a mean score of compliance with diet regimen and its all aspects among control and intervention groups (*p* < 0.05). Assessment of the effect of intervention on status of each dimension showed that mean and standard deviation of compliance with diet regimen and its aspects were increased after intervention and were statistically significant with a confidence level of 95% (*p* = 0.001), but the patients in the control group before and after the intervention had no significant difference in none of the final dimensions and constructs of compliance with diet regimen (*p* > 0.05) (Table 2).

**Table 2:** Mean and standard deviation of the variables in the control and intervention groups before and after the intervention

Variables	Stages	Mean ± SD		*P*.value
		Intervention group	Control group	
making effort for treatment	Before intervention	25.7 ± 3.7	25.5 ± 4.4	0.76^a^
	After intervention	43.5 ± 3.07	26.9 ± 5.3	0.001^a^
	*P*.value	0.001 ^b^	0.18 ^c^	–
intention to take the treatment	Before intervention	17.57 ± 3.14	17.3 ± 3.34	0.75^b^
	After intervention	31.97 ± 2.58	17.17 ± 3.01	0.001^b^
	*P*.value	0.001 ^b^	0.84 ^b^	–
adaptability	Before intervention	19.7 ± 2.5	20.6 ± 2.3	0.16^a^
	After intervention	30.3 ± 3.9	19.5 ± 2.5	0.001^a^
	*P*.value	0.001 ^c^	0.14 ^c^	–
integrating illness into life	Before intervention	16.2 ± 2.4	16.5 ± 4.5	0.75^a^
	After intervention	21.8 ± 2.3	16 ± 2.9	0.001^a^
	*P*.value	0.001 ^c^	0.61 ^b^	–
stick to the treatment	Before intervention	11.6 ± 2.45	11.4 ± 2.26	0.70^d^
	After intervention	16.4 ± 2.6	11.43 ± 2.48	0.001^d^
	*P*.value	0.001 ^b^	0.94 ^b^	–
commitment to treatment	Before intervention	16.8 ± 3.3	16.7 ± 2.8	0.90^a^
	After intervention	20.4 ± 5.5	16.8 ± 2.7	0.001^d^
	*P*.value	0.003 ^c^	0.68 ^c^	–
indecisiveness for applying treatment	Before intervention	12.53 ± 2.01	12.5 ± 2.04	0.95^d^
	After intervention	15.13 ± 2.08	13.20 ± 2.29	0.001^a^
	*P*.value	0.001 ^c^	0.05 ^b^	–
compliance	Before intervention	120.5 ± 6.8	120.6 ± 10.6	0.97^a^
	After intervention	179.7 ± 9.6	120.5 ± 8.2	0.001^a^
	*P*.value	0.001 ^b^	0.96 ^b^	–

*P*.value significant at *P* < 0.05

*SD* Standard deviation

^a^Mann-Whitney U

^b^Paired-samples t-test

^c^Wilcoxon Test

^d^Independent t-test

Status of compliance with a diet regimen of the patients was at the medium level before training in two control and intervention groups, but after the intervention of family-centered empowerment, the status of compliance with the diet regimen of the patients in the intervention group was good and status of the patients in the control group was moderate (Table 3).

**Table 3:** Distribution of partial and absolute frequency of compliance in intervention and control groups before and after intervention

Variables	Stage	Dimensions	Intervention group *n* (%)	Control group *n* (%)
compliance	Before intervention	Weak	–	–
		Mean	2 (66.7)	18 (60)
		Good	10 (33.3)	12 (40)
		Very Good	–	–
	After intervention	Weak	–	–
		Mean	–	19 (63.3)
		Good	23 (76.7)	11 (36.7)
		Very Good	7 (23.3)	

## Discussion

The results of the study showed that the implementation of the family-oriented empowerment model by using telephone follow-up had a positive effect on increasing patients’ adherence to the treatment regimen.

The results showed that the two groups before intervention were equal regarding the score of compliance with the diet regimen. Compliance with the diet regimen of the patients in the intervention group was increased before intervention compared to post-intervention, but regarding the control group, diet therapy of the patients in the control group before intervention did not change significantly compared to post-intervention. All the patients in the intervention group had a higher level of compliance with diet regimen after the intervention compared to the control group. In the study by Ghotbi et al., [23] after training in the intervention group, a total score of self-care behaviors and their aspects was increased which this difference was statistically significant in line with the current study. In the clinical trial study conducted by Azhdari Mamaghani et al. [24] on 156 patients with type 2 diabetes, the results showed that the difference in self-efficacy score in the two intervention groups was significant before and after the intervention and, no difference was observed in the control group. Between the two intervention groups, the results showed that self-efficacy was higher in the telenursing empowerment group. The reduction of HbA1c was significant only in the telenursing empowerment group. These results showed that performing training methods based on empowering the patient which is done by cooperation and centering patient and his/her family, can play important role in obtaining correct health behaviors and achieving independence of the patients with diabetes and/or other chronic diseases in self-caring. The study by Turner et al., [25] showed that long-distance care of patients with diabetes mellitus type 2 through telephone follow-up by a nurse increases compliance with diet regimen by the patients. In addition, Aggarwal et al., [26] in their research concluded that the compliance with diet regimen rate of the patients without the appropriate presence of their family was significantly decreased after discharge. Considering training of the patient’s family can facilitate appropriate management and control in them. Family-centered training and follow-up with the aim of awareness reinforcement, improvement of performance and attitude in supporting of patients with diabetes mellitus type 2 who have problems in compliance with diet regimen are very effective. Results of the study by Zakerimoghadam et al., 2010 showed a significant difference among control and intervention groups regarding diet, medication regimen, exercise training, foot care, supervision on blood sugar and medication use. In addition, after 3 months, HbA1c hormone significantly differed in two groups. Telephone follow-up by the nurse was effective in the improvement of compliance with diet regimen by the patients with diabetes in the study by Zakerimoghadam et al., 2010 [27] which is consistent with the current study. Rezai Asl et al., [28] showed that compliance with diet regimen at three stages of pre-intervention and post-intervention and 4 weeks after intervention in case group differed significantly, and they showed that performing family-centered empowerment pattern can be effective in compliance with diet regimen and increases compliance with diet regimen which is in line with the current study. Shahsavari and Bavarsad [29], in a study with the aim of examining the effectiveness of telenursing on illiterate diabetic patients aged 50 years and older, showed that HbA1c decreased significantly among the patients of intervention group who received nurses-led telephone follow-up and their adherence to treatment plan increased. The study by Kamrani et al., [30] showed that the mean score of compliance with diet regimen in the two groups before intervention was significant regarding post-intervention which is in line with the current study.

No significant change was observed after the interventional training in blood sugar control in patients with diabetes in the study by Jalilian et al., [31], and it seems that other factors such as level of literacy and cultural factors affect blood sugar control. The study population in the mentioned study was rural and its participants had an elementary education degree which was not in line with the current study. Results of the study by Wong et al., [32] showed that telephone follow-up might not be effective in reducing readmission of patients, and there is a need to use various methods to train after discharge. This study inconsistent with our study states that all the points which the patient should be aware of them must first be trained and along with training, efforts such as telenursing should be done, while in the current study, most information needed by the patient was firstly asked and recorded in his/her file, and then was performed as an arranged program of training through telenursing.

Lack of establishment of mental and emotional relationship with patients and family members which it is needed to establish effective relationships among participants and diet therapists and to prevent it and stating the importance of the issue, and difference in culture, motivation, and personal interests of the family which requires assessment and recognition of the public culture of the region by the researchers were all the limitations of the study.

## Conclusions

The findings showed that diabetic patients are at an undesirable level regarding compliance with diet regimen, and empowerment of the patient in an environment of the family is one of the useful approaches in training of the diabetic patients. Given the effectiveness of the intervention performed to improve the status of compliance with diet regimen, training of telenursing based on family-centered empowerment pattern in regard with improve the status of compliance with diet regimen in patients with diabetes is applicable and possible. Therefore, applying family-centered patterns in health policy-making related to chronic diseases can play important role in increasing the health level of society. It is recommended to conduct this study on a wider scale and in other hospitals and other types of diabetic patients and assess the results.

## Data Availability

The datasets used and analyzed during the current study are available from the corresponding author on reasonable request.
